# Proceedings of Patient Reported Outcome Measures (PROMs) Conference Birmingham 2018: Birmingham, UK. 20 June, 2018

**DOI:** 10.1186/s41687-018-0087-9

**Published:** 2018-12-18

## Introduction

### I1 JPRO proceedings: PROMs research conference, University of Birmingham, 20^th^ June 2018

#### Elizabeth Gibbons^3^, Melanie Calvert ^4,5^, Jennifer Bostock^1^, Magdalena Skrybant^2^

##### ^1^University of Oxford, Oxford, United Kingdom; ^2^University of Birmingham, Birmingham, United Kingdom; ^3^Health Services Research Unit, Nuffield Department of Population Health, University of Oxford; ^4^Centre for Patient Reported Outcomes Research, Institute of Applied Health Research, University of Birmingham, Edgbaston, Birmingham, UK B15 2TT; ^5^NIHR Birmingham Biomedical Research Centre and NIHR Surgical Reconstruction and Microbiology Research Centre, University Hospitals Birmingham NHS Foundation Trust and University of Birmingham B15 2TT

Following the success of the last two PROMs Research Conferences held at University of Sheffield (2016), St Anne’s College, University of Oxford, (2017) we report the proceedings of the 2018 conference hosted by the Centre for Patient Reported Outcomes Research at the University of Birmingham, UK on 20th June 2018.

**Aims of the conference:**

The aim of the conferences was to bring together leading international experts, clinicians, patient partners and early career researchers to engage with the latest advances in the field of PROMs research and implementation.

**Acknowledgements**

Key funding and support was provided by the following NIHR Collaborations for Leadership in Applied Health Research and Care (CLAHRC): Oxford, Yorkshire & Humber, West Midlands, West and East of England. Further support was given from West Midlands Academic Health Science Network and the conference was endorsed by the International Society for Quality of Life Research (ISOQOL).

The important role of patients and the public in planning, attending and presenting at the Conference was acknowledged through the ‘Patients Included’ Chartermark.

Oral and poster abstracts were encouraged from clinicians, researchers, patient partners, industry, SMEs and others working in the field. Several presentation prizes were awarded.

**Plenaries**

There were two stimulating plenary sessions: PROs from a regulatory and patient perspective and the evolution of Patient and Public Involvement in PRO research.

**Dr Daniel O’Connor** from the MHRA discussed the importance of incorporating the patient experience throughout the drug development process and outlined several international collaborations aiming to gain consensus on PRO methodology such as SPIRIT-PRO and SISAQOL.

**Dr Tessa Richards**, senior editor of the BMJ, carer and cancer patient presented a powerful narrative of how challenging it is for patients to make decisions about treatments in the absence of patient outcomes and experiences information.

**Dr Kirstie Haywood**, Lead of the PRO Research programme, University of Warwick provided a detailed overview of the advances in Patient and Public Involvement in PRO research. Current guidance could facilitate co-production of future PRO research.

**Funding**

Gibbons is funded by the National Institute for Health Research (NIHR) Collaboration for Leadership in Applied Health Research and Care Oxford at Oxford Health NHS Foundation Trust. The views expressed are those of the author(s) and not necessarily those of the NHS, the NIHR or the Department of Health and Social Care.

Calvert is funded by the NIHR Birmingham Biomedical Research Centre and the NIHR Surgical Reconstruction and Microbiology Research Centre at the University Hospitals Birmingham NHS Foundation Trust and the University of Birmingham. The views expressed are those of the author(s) and not necessarily those of the NHS, the NIHR or the Department of Health.

**Oral presentations**

There were a large number of abstract submission: n=115; following peer review, n=36 oral sessions’ and n=79 posters.

There was a huge emphasis the inclusion of PROs in innovative methods and digital technologies ranging from the evaluation of digital capture to inclusion of PROs in platforms to support self-management.

*PROs in chronic diseases/different settings* **.**Presentations included the use of PROs in primary care and A&E, specific conditions: inflammatory conditions, musculoskeletal and chronic pain as well as cancer, joint replacement, and rare conditions.

*Cutting edge methods*. A broad range of novel statistical, evaluations of innovations and approaches to capturing PROs from specific populations such as carers and patients with dementia and impact of PROs in clinical trials

*Pushing boundaries* included examples of patient and public involvement in research in terms of outlining good practices and frameworks for meaningful PPI, co-design of PROs

*Digital capture* Presentations illustrated the emergence of digital technology and innovations to include PROs to capture symptoms and adverse events; evaluation of paper and electronic administration and the online development of PROs to support self-management. Digital approaches for specific conditions were also presented: orthopaedics, kidney disease and trauma.

Further oral presentations were presented under the Minimising PRO waste theme and Economic Evaluation.

152 delegates attended including 5 patients.

**PROMs includes Patients!**

**Magdalena Skyrbant: PPI/E Lead CLAHRC West Midlands**

For the first time, the PROMs Conference was awarded the ‘Patients Included’ Chartermark. Patient/public partners were valued members of the Conference Organising Committee and their contributions influenced the content and delivery of the event. In particular, patient/public partners wanted the Conference to be an opportunity to underline the importance of involving patients in PROs development, share best practice, and explore new ideas/strategies. This was achieved through: Plenary Discussions, which offered both patient and academic perspectives on patient involvement in PROs research; a dedicated panel on ‘Pushing the Boundaries of Patient and Public Involvement (PPI) in PROs research’; and presenting an award to the research team demonstrating the ‘Best PPI’. The Conference was co-chaired by patient partner, Gary Price, which underlined the clear commitment from the Conference Organisers to public involvement in PROs research.

In addition to planning and delivering the PROMs Conference, the Organising Committee were keen to incorporate insights and perspectives from patients during the event. 10 bursaries were offered to patients/public (5 delegate/5 delegate + travel) to enable patients and the public to attend and participate in the event and present issues most important to patients.

A measure of how successful PROMs 2018 included patients is the integration of patients and the public at the event. Both during and in between sessions, patient and public delegates mingled effortlessly with researchers. There were opportunities in the Conference for patients and the public to discuss current research, challenge ideas, and influence avenues for future exploration.

It is hoped that the next PROs Conference will build on the success of including patients and that more patients will attend, ensuring that all future PROMs research benefits from the rich contributions patients can offer to make research more relevant and more successful.

**Jennifer Bostock: Public Advisor, Nuffield Department of Population Health at the University of Oxford**

I am a patient/public research advisor to the Nuffield Department of Population Health at the University of Oxford where I have been working on various projects for a number of years. Amongst these projects is number involving PROMS of one kind or another. My interest in PROMS (apart from the marvel that is the BBC concert series) stems from my experience as a patient and carer completing these questionnaires whilst in waiting rooms, consultations and post consultation. It has intrigued me what happens to the information I give on the questionnaires and what is the point of them. It is the answer to these questions that I had in mind when I attended the 2018 PROMS conference.

The conference took place in the splendour of the University of Birmingham’s Great Hall, my first trip, but hopefully not my last. The conference was very well organized, well attended and the presentations and stalls were varied, interesting and inviting with lots to interest academics, clinicians & patients.

I was there to watch, listen, enjoy and to see if my questions were answered. Were they? To an extent yes, but what us patients have to remember about academic conferences is, that although the subject might be ‘patient orientated’ that is that PROMS are ultimately about helping improve ‘outcomes’ for us patients, conferences are really places run by and for academics. Hence some of the presentations were somewhat esoteric, falling into the spiral staircase that is PROMS methodology. But these were not designed to entertain or inform people like me, but what was were the presentations under the rather ambiguous title of ‘Pushing Boundaries’ where the audience were told about the ways in which patients/carers and members of the public have been involved in PROMS research. I was there to listen but also as part of a lay judging panel and so I listened with great attention, to the style, content and delivery all of which were different but interesting in their own ways. The one that stood out for me and became the winner in the category, being judged unanimously by the panel, was Grace Turner and Gary Price (patient partner). It wasn’t so much the clear commitment, enthusiasm and value placed on PPI which was evident both in this presentation and indeed in all, but the rather brave attempt to creatively involve the patient partner. This was most clearly demonstrated by the showing of a simple photograph, one with Gary and members of the research team at an engineering firm. Why such a photo? Because the patient partner is an engineer and he invited the researchers to see ‘how it’s done’ in his profession, and the researchers took the unusual step of accepting this offer. The result was an insight into a world outside academia, outside the clinical environment and into private sector engineering. Lessons learned, stories told and tips exchanged the research team not only had a jolly good day out but a host of ideas which they could put into research practice. This sharing of professional worlds is something I have been long advocating having worked in private and public sector diverse fields and recognizing that many academics have only ever worked in universities or for the NHS.

What is the recipe for such PPI success? 1 part willingness, 2 parts right people & 3 parts imagination, creativity and bravery. I found this an inspiring and engaging presentation on what might otherwise be a rather dry subject, for PROMS, no matter how important and useful they may be, are not the most tempting of subjects with which to inspire, but inspire this one did.

In sum the conference was an enlightening affair, interesting people, doing important work and in a superb setting – oh and there’s a pretty good art gallery next door which I also found myself being inspired by…..

**Presentation Prize Winners**

Several prizes were judged and awarded as follow:

Best Oral: Early Career research award: **Jenny Harris**, Handling missing PROs for multivariate models: a practical guide to multiple imputation using chained equations

Best Poster: Best PhD poster: **Sally Appleyard**, Barriers to remote electronic completion of Quality of Life Patient reported outcome measures: qualitative results from a feasibility study in men with advanced prostate cancer.

Oral or Poster: Best patient and public involvement award for the team which have demonstrated excellence and/or innovation in involving the public: **Grace Turner, Olalekan Lee Aiyegbusi, Derek Kyte, Anita Slade, Magdalena Skrybant, Gary Price, Melanie Calvert**, Embedding patient and public involvement within the Centre for, Patient Reported Outcomes Research.

## Theme: Plenary Speakers

### O1 PROs from the regulatory perspective

#### Dr Daniel O’Connor

##### Medicines and Healthcare products Regulatory Agency, London, United Kingdom

Incorporating the patient experience throughout the drug development process is of increasing interest and importance. However, poorly defined PRO objectives and methodology in regulatory submissions has traditionally hampered the usefulness of PROs in regulatory decision making. The UK’s Medicines and Healthcare products Regulatory Agency (MHRA) works with other member states (MS) and the European Medicines Agency (EMA) in a network where there is an extensive collection of scientific & regulatory guidance documents. Guidelines provide a basis for how MS/EMA interpret & apply the requirements for demonstration of quality, safety & efficacy in drug licensing.

A new appendix to the main anticancer guideline, use of patient-reported outcome (PRO) measures in oncology studies, was released in April 2016. This appendix outlines the broad principles of scientific best practice from a regulatory perspective, aiming to encourage developments in the methods and application of PROs. Here, the importance of the patient’s point of view on their health status is also fully acknowledged and may be used in drawing regulatory conclusions regarding treatment effects, in the benefit risk balance assessment or as specific therapeutic claims.

Alongside the PRO appendix, there are encouraging recent international collaborative efforts that aim to generate consensus on PRO methodology, for example the standardising of protocol items (SPIRIT-PRO) and analysis and presentation methods (SISAQOL). In addition, the UK Accelerated Access Review recommendations state that ‘patients should be involved in horizon scanning and prioritisation, and this involvement should continue along the whole innovation pathway’. The impact of the appendix and these collaborative efforts are discussed in the context of evolving regulatory science and patient access initiatives.

### O2 ‘Working together to improve outcomes research’: how innovations from patient and public involvement (PPI) research can inform the future of patient-reported outcomes (PRO) and health-related quality of life (HRQoL) research

#### Kirstie Haywood

##### University of Warwick, Coventry, United Kingdom

**Objective**

To explore how advances in Patient and Public Involvement (PPI) research can inform patient-reported outcomes (PRO) and health-related quality of life (HRQoL) research.

**Background**

The policy drive for patient and public involvement (PPI) in health research has been growing for almost 20-years [ref]. In 2005, PPI became a statutory part of England’s national research governance framework, and in 2015 the rationale and value of PPI was highlighted in the Department of Health’s report ‘Going the Extra Mile’ (‘Breaking Boundaries’) commissioned by the Chief Medical Officer [ref]. Policy guidance endorses the importance of including PPI representation at all stages of the research journey, but guidance for how to do this is less common. However, recent years have witnessed several large programmes of work which are transforming the co-production of guidance to inform good PPI practice.

## Theme: Minimising PRO waste

### O3 Development of a core outcome set capturing key concepts relevant to safe and efficient evaluation of innovative invasive procedures

#### Kerry Avery, Shelley Potter, Nicholas Wilson, Rhiannon Macefield, Rob Hinchliffe, Sian Cousins, Natalie Blencowe, Daisy Elliott, Barry Main, Angus McNair, Jane Blazeby

##### University of Bristol, Bristol, United Kingdom

**Background**

Methods for introducing invasive procedures with/out a device (IP&D) into clinical practice vary. This contrasts with the highly-regulated environment for introducing pharmaceutical products. Consequences of insufficient outcome evaluation can be severe, and has resulted in scandals including recall of counterfeit spinal-fusion screws (US), removal of silicone breast implants (Europe) and concerns over vaginal mesh implants (UK). Clinicians and manufacturers are often free to choose how outcomes are assessed and reported. Heterogeneity prevents data syntheses and delays identification of problematic or promising innovations. Development of a core outcome set (COS) to measure and report in all early phase studies (EPS), accompanied by transparent mandated reporting guidelines, is necessary to introduce innovative IP&D safely and efficiently.

**Aim**

To describe methods and early results (steps 1-2) of the identification and characterisation of outcome domains for minimal reporting of innovative IP&D.

**Methods**

1. Document analysis to explore how regulatory bodies describe how outcomes are selected/measured/reported;
2. Content analysis of EPS and IP&D reporting systems to identify and characterise generic innovation-related outcome domains;
3. Refine conceptualised domains within novel IP&D case studies;
4. Iteratively engage stakeholders (surgeons, patients, manufacturers, regulators) to develop reporting guidelines.

**Results**

Regulatory bodies overseeing new IP&D include the UK Medicines and Healthcare products Regulatory Agency and Interventional Procedures Advisory Committee, European CE Marking, European Medicines Agency and US Food and Drug Administration. No single system provides detailed mandated guidance on outcomes relevant to comprehensively evaluate innovative IP&D. Early findings indicate that broader conceptualisation of outcomes, beyond traditional AEs and complications, is needed, including:

- Innovation delivered with intended/unintended effect;
- Innovation abandoned (intra/postoperatively);
- Innovation modified;
- Innovation associated with longer-term unintended/unanticipated effects.

**Conclusions**

Robust methods for early phase evaluation of innovative IP&D are urgently needed and identifying core outcome domains to inform selection of agreed outcomes for IP&D is the first necessary step.

### O4 Current state of statistical analysis of patient reported outcomes data in cancer randomized controlled trials on locally advanced and metastatic breast cancer – a systematic review

#### Madeline Pe^1^, Lien Dorme^1^, Corneel Coens^1^, Ethan Basch^2^, Melanie Calvert^3^, Alicyn Campbell^4^, Charles Cleeland^5^, Kim Cocks^6^, Laurence Collette^1^, Linda Dirven^7,8^, Amylou C Dueck^9^, Nancy Devlin^10^, Hans-Henning Flechtner^11^, Carolyn Gotay^12^, Ingolf Griebsch^13^, Mogens Groenvold^14^, Madeleine King^15^, Michael Koller^16^, Daniel C Malone^17^, Francesca Martinelli^1^, Sandra A Mitchell^18^, Jammbe Z Musoro^1^, Kathy Oliver^19^, Elisabeth Piault-Louis^4^, Martine Piccart^20^, Francisco L Pimentel^21,22^, Chantal Quinten^23^, Jaap C Reijneveld^24^, Jeff Sloan^25^, Galina Velikova^26^, Andrew Bottomley^1^

##### ^1^EORTC, Brussels, Belgium; ^2^University of North Carolina, Chapel Hill, NC, USA; ^3^University of Birmingham, Birmingham, United Kingdom; ^4^Genentech, San Francisco, CA, USA; ^5^University of Texas MD Anderson Cancer Center, Houston, TX, USA; ^6^Adelphi Values, Bollington, Cheshire, United Kingdom; ^7^Leiden University Medical Center, Leiden, Netherlands; ^8^Haaglanden Medical Center, The Hague, Netherlands; ^9^Mayo clinic, Scottsdale, AZ, USA; ^10^Office of Health Economics, London, United Kingdom; ^11^University of Magdeburg, Magdeburg, Germany; ^12^University of British Columbia, Vancouver, Canada; ^13^Boehringer-Ingelheim, Frankfurt, Germany; ^14^University of Copenhagen, Copenhagen, Denmark; ^15^University of Sydney, Sydney, Australia; ^16^University Hospital Regensburg, Regensburg, Germany; ^17^University of Arizona, Tucson, USA; ^18^National Cancer Institute, Bethesda, MD, USA; ^19^International Brain Tumour Alliance, Surrey, United Kingdom; ^20^Institut Jules Bordet, Université Libre de Bruxelles (ULB), Brussels, Belgium; ^21^Blueclinical, Porto, Portugal; ^22^Centro de Estudos e Investigação em Saúde da Universidade de Coimbra, Coimbra, Portugal; ^23^European Centre for Disease Prevention and Control (ECDC), Stockholm, Sweden; ^24^VU University Medical Center, Amsterdam, Netherlands; ^25^Mayo Clinic, Rochester, MN, USA; ^26^University of Leeds, Leeds, United Kingdom

*For the Setting International Standards in Analyzing Patient-Reported Outcomes and Quality of Life Endpoints Data (SISAQOL) Consortium*’

**Background**

Although patient reported outcomes (PROs) such as health-related quality of life (HRQOL) are important endpoints in randomized controlled trials (RCTs), there is little consensus about analysis, interpretation and reporting of these data. A systematic review was conducted to assess variability, quality and standards of PRO data analyses in advanced breast cancer RCTs.

**Methods**

We searched PubMed for English language articles in peer-reviewed journals between January 2001 and October 2017. Eligible papers reported PRO results from RCTs involving adult advanced breast cancer patients receiving anti-cancer treatments. A sample size of at least 50 patients was required.

**Results**

Sixty-six RCTs met the inclusion criteria. Only a small number of RCTs included a specific PRO research hypothesis (8/66, 12%). There was heterogeneity in statistical methods used to analyze PRO data, across a range of longitudinal and cross-sectional techniques. Not all articles addressed the problem of inflated type I error resulting from multiple testing: only 27 of 66 trials (41%) statistically corrected for multiple items/scales or assessed only one relevant item/scale; 41 of 66 trials (62%) either statistically corrected for independent tests of multiple assessments, used a statistical technique that took into account repeated assessments, or analyzed only one follow-up assessment. Fewer than half of RCTs reported the clinical significance of their findings (28/66, 42%). The majority of trials did not report how missing data was handled (48/66, 73%).

**Conclusion**

Our review demonstrates a need to improve standards in analysis, interpretation and reporting of PRO data in cancer RCTs. Lack of standardization makes it difficult to draw robust conclusions and compare findings across trials. The Setting International Standards in Analyzing Patient-Reported Outcomes and Quality of Life Data (SISAQOL) Consortium addresses this need and has convened a series of working groups to develop recommendations on the analysis of PRO data in RCTs.

### O5 Handling missing PROs for multivariate models: a practical guide to multiple imputation using chained equations

#### Jenny Harris^1,2^, Victoria Cornelius^3^, Ed Ed Purssell^2^, Emma Ream^1^, Jo Armes^1^

##### ^1^University of Surrey, Guildford, United Kingdom; ^2^King's College London, London, United Kingdom; ^3^Imperial College London, London, United Kingdom

**Background**

Missing PRO data in the field of psycho-social oncology is common, particularly with repeated measures and longitudinal designs. Despite numerous simulation studies recommending otherwise, case-wise deletion, mean or single imputation are widely used but may reduce available data and lead to biased estimates. In many circumstances, Multiple Imputation using Chained Equations (MICE) followed by pooling using Rubin’s rule will be appropriate but is still not widely implemented in practice.

**Aim**

To describe key practical stages in using multiple imputation using chained equations (MICE) for the development of multivariate models from the field of psychosocial-oncology, drawing together best practice research and current guidance.

**Methods**

We outline key stages of MICE, with a worked example using data from a longitudinal sample of women treated for breast cancer. This data incorporates several PROs, including the widely used Hospital Anxiety and Depression Scale (HADS).

**Results**

Stages of MICE include deciding to impute (including assessing extent and patterns of missingness, associations with missingness and assessing mechanisms for missingness); imputing different types of PRO (continuous, binary, categorical; skewed); specifying imputation models (variable selection and model form); deciding on the number of imputations; and checking model convergence. Specific techniques relating to how variable selection can be used with MICE data will be described, as will common issues and challenges in implementing MICE.

**Conclusions**

MICE programmes are available in many statistical packages but are underutilised with complete-case analysis common. Wider knowledge and understanding of MICE’s practical implementation, beyond statisticians and data managers, may improve multivariate model development using PRO.

## Theme: PROS in chronic disease/different settings

### O6 A survey of Patient Reported Outcome Measures (PROMs) used in General Practice in England

#### Grace Turner^1^, Sam Finnikin^1^, Tom Keeley^2^, Derek Kyte^1^, Clare Taylor^3^, Helen Stokes-Lampard^4^, Melanie Calvert^1^

##### ^1^University of Birmingham, Birmingham, United Kingdom; ^2^GSK, Uxbridge, United Kingdom; ^3^University of Oxford, Oxford, United Kingdom; ^4^Royal College of General Practitioners, London, United Kingdom

**Background**

Patient-reported outcome measures (PROMs) can provide valuable information from the patient perspective on the impact of disease and treatment on symptoms and quality of life. Whilst PROMs are used extensively in a clinical research setting there is increasing interest in the use of PROMs in a routine clinical setting: i) at an individual level to facilitate communication, shared-decision making and symptom management and; ii) at an aggregate level for audit and benchmarking purposes. The aim of this research was to evaluate the current use of PROMs in routine general practice and explore barriers and facilitators to their implementation.

**Methods**

An electronic self-completed questionnaire was administered to 100 English general practitioners via an online doctor’s community. Ethical approval was provided by the University of Birmingham Ethical Review Board.

**Results**

One hundred general practitioners from across England responded. Twenty three reported that they do not use PROMs in clinical practice. Of the PROMs use reported, 35% were in mental health, 15% for urology and 10% were for sleep apnoea; however, 15% of tools GPs reported using were not PROMs. The most cited reasons for using PROMs were as an aid to clinical management (66%) and as a screening or diagnostic tool (61%). Time constraints and compulsory completion were reported as the biggest barriers to engaging with PROMs in clinical practice. GPs would prefer to use PROMs that are well integrated with their clinical systems.

**Discussion**

There is a misunderstanding amongst many GPs as to what constitutes a PROM and their use in practice appears limited. However, there are some tools which have gained traction indicating a willingness to integrate PROMs into clinical practice. Tools that aid diagnostics and management that are integrated with clinical systems may be most likely to be used.

### O7 Fatigue in patients with newly presenting inflammatory arthritis and inflammatory arthralgia

#### Gurpreet Jutley^1,2^, Ilfita Sahbudin^2,1^, Peter Nightingale^3^, Kalvin Sahota^1^, Christopher Buckley^1,4^, Amy Livesey^4^, Stephen Young^1^, Andrew Filer^2,1^, Karim Raza^1,4^

##### ^1^Rheumatology Research Group, Birmingham, United Kingdom; ^2^University Hospitals Birmingham NHS Foundation Trust, Birmingham, United Kingdom; ^3^Wolfson Computer Laboratory, Queen Elizabeth Hospital Birmingham, Birmingham, United Kingdom; ^4^Sandwell & West Birmingham Hospitals NHS Trust, Birmingham, United Kingdom

**Background**

Fatigue is a common and troublesome symptom of inflammatory arthritis but the factors influencing its development and magnitude are not well understood. We assessed the extent of fatigue in patients with newly presenting inflammatory arthritis and inflammatory arthralgia and its relationship with clinical and demographic variables.

**Method**

The Birmingham Early Arthritis Cohort is a prospective cohort of patients with newly presenting, DMARD naive inflammatory arthritis or inflammatory arthralgia. Sociodemographic and clinical data, including fatigue (FACIT-F and VAS-F), disability (HAQ) and mood (PHQ9), were collected at baseline and follow up after at least 12 months.

**Results**

369 patients (65.6% female, age 50.8 +/- 14.8 years (mean +/- SD)) were included, 295 (79.9%) with inflammatory arthritis and 74 (20.1%) with inflammatory arthralgia. Fatigue was common and levels of fatigue equivalent in patients with inflammatory arthralgia and arthritis. Multivariate analysis revealed that low mood and disability were independently associated with fatigue in both patient groups, with tender joint count, additionally, independently associated with fatigue in inflammatory arthritis patients at baseline. Multinomial logistic regression modelling revealed low mood and increased pain were associated with worse FACIT-F at follow up and improvement of DAS28 ESR (≥1.2) was associated with an improved FACIT-F at follow up.

**Conclusions**

Fatigue is comparable in magnitude in the early stages of inflammatory arthritis and inflammatory arthralgia. Conventional clinical and demographic variables do not fully explain its variability. Further research is required to investigate the mechanisms underlying fatigue in early inflammatory joint disease.

### O8 Models used for risk-adjustment of health outcomes in musculoskeletal services: a systematic review of the literature

#### Roanna Burgess^1,2^, Annette Bishop^1^, Martyn Lewis^1^, Jonathan Hill^1^

##### ^1^Research Institute for Primary Care and Health Sciences: Keele University, Stoke on Trent, United Kingdom; ^2^Sandwell and West Birmingham Hospitals NHS Trust, Birmingham, United Kingdom

**Background**

Risk-adjustment is an established method to take account of variations across cohorts in baseline patient factors, when comparing health outcomes. Although commonplace, there is a lack of evidence as to the most appropriate risk-adjustment model to use, to enable fair comparisons of musculoskeletal health outcomes.

**Aim**

To conduct a systematic review summarising evidence of the development, validation, and performance of musculoskeletal risk-adjustment models, and to make recommendations for future risk-adjustment methodology.

**Methods**

Data Sources

Searches included; AMED, CINAHL, EMBASE, HMIC, MEDLINE, and the grey literature.

Eligibility Criteria

Studies; from 1992, English language, musculoskeletal adult population, developing or validating a risk-adjustment model, using a relevant patient reported outcome measure (PROM), and feasible for clinical collection.

Data Synthesis

Two reviewers evaluated selected papers. The CASP Cohort Tool was used to assess quality.

**Results**

14 studies were included; eight US studies on the Focus on Therapeutic Outcomes (FOTO) model (pooled n=546,726 patients (with pre/post treatment data)) and six UK studies related to the UK National PROMs Programme model (pooled n=282,424 patients (with pre/post treatment data)). The majority used retrospective data, restricted to complete datasets. Both US and UK models showed good predictive ability (R2 18-42%). There was significant methodological crossover. Common model variables were; baseline PROM score, age, sex, comorbidities, symptom duration, and surgical history. Reduced quality scores were mainly due to acceptability of patient recruitment, and completeness and length of patient follow up.

**Conclusion**

Two musculoskeletal risk-adjustment models were identified with similar predictive performance. Recommendations for future practice are provided. Effective risk-adjustment modelling across musculoskeletal clinical pathways of care will allow for further development of performance profiling and benchmarking across musculoskeletal practice, with the aim of improving quality and equity of musculoskeletal healthcare.

### O9 Patient-reported outcome measures (PROMs) in clinical practice for non-malignant pain: a realist review and theoretical framework

#### Michelle Holmes^1,2^, Felicity Bishop^1^, Dave Newell^2^, Jonathan Field^3^

##### ^1^University of Southampton, Southampton, United Kingdom; ^2^AECC University College, Bournemouth, United Kingdom; ^3^Back2Health, Portsmouth, United Kingdom

**Background**

The use of patient-reported outcome measures (PROMs) has increasingly been incorporated into routine clinical practice. Research to date suggests that PROMs may affect the process and outcome of care. The theoretical basis underpinning the use of PROMs in clinical practice remains underdeveloped; much of the published research has focused on the impact PROMs may have in clinical practice with limited research to understand the potential mechanisms behind any effects. The aim of this realist review was to identify the processes by which PROMs might influence health outcomes in routine clinical practice for non-malignant pain.

**Methods**

An electronic search was carried out of relevant databases: MEDLINE, EMBASE, PsycINFO, PsycARTICLES, Cochrane Library and Web of Science. The review examined reviews, letter, editorials and commentaries in order to identify theories and critical pieces of literature exploring how PROMs feedback might work in routine clinical practice. Text from 61 relevant papers was included and coded inductively. Codes were examined for patterns; to form a preliminary conceptual explanation of the processes and mechanisms of actions when using PROMs. Findings were reviewed in relation to formal psychological theories and empirical literature, and a theoretical framework was developed.

**Results**

The review suggests that PROMs may affect patients through various processes: incorporating increasing clinician knowledge, facilitating patient-doctor interaction, provision of patient-centered care, monitoring, informing strategies to improve care, therapeutic relationship, patient satisfaction, patient behaviour and factors which influence clinicians’ use of PROMs. The developed a novel theoretical framework: The Patient Reported Outcome Measures Pathway Theory (PROMPT).

**Conclusions**

The findings of this realist review highlight a series of processes by which PROMs may influence patient outcomes within the context of treating non-malignant pain. PROMPT provides a valuable foundation to guide future research on the use of PROMs and the processes by which PROMs may influence health outcomes.

### O10 How do alcohol-dependent individuals, who repeatedly present to A&Es, engage with structured questionnaires? A mixed methods study

#### Agnes Williams

##### ICON plc, London, United Kingdom. King's College, London, London, United Kingdom

**Background**

Alcohol abuse is a significant burden to Accident and Emergency departments (A&Es) where frequent visitors contribute to overcrowding and delays. Limited research has explored the attributes of Alcohol Frequent Attenders (AFAs) as there seem to be challenges when conducting quantitative studies with them. This study investigated how alcohol-dependent individuals who frequently visit A&Es engage with commonly used questionnaires, to explore possible validity issues with the scales when used in this patient population.

**Methods**

This study used existing data collected for a previous research with alcohol frequent attenders of A&Es (n=30). Mixed methods research was used to address the aims. The quantitative component assessed the scores of the following scales: the EuroQol health questionnaire (EQ-5D-3L), the Severity of Alcohol Dependence Questionnaire (SADQ) and the Alcohol Problems Questionnaire (APQ); the qualitative component analysed the audio recordings of the administration of these instruments.

**Results**

Quantitative results presented various health issues, with third of the participants reporting severe pain/discomfort and anxiety/depression in the EQ-5D, over 50% had severe alcohol dependence according to the SADQ, and various alcohol related problems were measured by the APQ. While the overall engagement with the questionnaires was good, the qualitative analysis revealed some problems with each. The visual analogue scale (VAS) of the EQ-5D was commonly misunderstood. The instructions of the “reinstatement of drinking” and the “quantity/frequency” subscales of the SADQ presented challenges for the respondents, suggesting possible validity problems with the instrument. The APQ had one item where the wording was confusing.

**Conclusion**

Findings highlight the necessity to review commonly used, existing questionnaires involving target patient groups. This might help to increase the content validity of these questionnaires and contribute to gain more accurate knowledge about alcohol frequent attenders. Further investigation is recommended using in-depth, cognitive interviewing.

### O11 What outcomes matter in cancer? A literature review

#### Amanda Cole^1^, Patricia Cubi-Molla^1^, Miaoqing Yang^2^, Jack Pollard^2^, Jon Sussex^2^, Paula Lorgelly^1^

##### ^1^Office of Health Economics, London, United Kingdom; ^2^RAND Europe, Cambridge, United Kingdom

**Background**

There have been unprecedented medical advances in cancer treatments globally, but securing access to those treatments can be problematic, and the UK often lags behind other high income countries. A number of schemes and policies have been implemented to try and address the issue (e.g. Cancer Drugs Fund, Early Access Scheme, Accelerated Access Review), with varying degrees of success. Increasingly, outcome-based reimbursements are suggested as a way to directly link payment with a patient’s outcomes, thereby reducing uncertainty for payers and improving patient access. A fundamental first step which is often overlooked is: what outcomes are valued?

**Aim**

To understand, from previously published research, what outcomes are important to patients?

**Methods**

We undertake a rapid evidence assessment of the literature. We consider peer reviewed academic papers and the grey literature. Outcomes identified are organised according to whether they represent clinical measures, social outcome measures (e.g. return to work), patient reported outcome measures (PROMs) including patient reported experience measures, or other patient defined measures.

**Results and discussion**

Given the hypothesis that our health care system should deliver value and thus pay for value, understanding what is of value to patients in terms of cancer outcomes is an important step in operationalising outcomes-based reimbursement. This review of the literature provides the necessary grounding in the plethora of available options to measure outcomes, and their relative merits. This will inform the next step of our research: to explore and confirm with patients what really matters, and investigate how the health system can reflect this in choosing when and how to fund treatments.

### O12 Comorbidity in patient-report and administrative data varied in agreement in a cohort of joint replacement patients

#### Belene Podmore^1,2^, Andrew Hutchings^1,2^, Jan van der Meulen^1,2^

##### ^1^London School of Hygiene & Tropical Medicine, London, United Kingdom; ^2^Royal College of Surgeons of England, London, United Kingdom

**Background**

Studies of epidemiology most commonly draw on administrative data for casemix adjustment for comorbidities. Increasingly, self-reported survey data has also been used as a source of data on comorbidities. Few studies however, have explored the accuracy of self-reported comorbidity compared to comorbidity recorded in administrative data.

**Aim**

To examine the level of agreement between patient-reported comorbid conditions and hospital administration records measures of comorbidity in patients undergoing hip or knee replacement surgery in the English NHS.

**Methods**

Patient-report survey data from 676,428 patients in 2009-2016 via the Patient Reported Outcome Measures (PROMs) programme was linked to inpatients Hospital Episode Statistics (HES) admissions data. Levels of agreement were compared for 11 comorbidities using Cohen’s kappa, sensitivity and specificity. Sensitivity analysis was conducted by length of look-back period and comorbidity sub-type.

**Results**

Specificity was high (>90%) for all 11 comorbidities. However, sensitivity varied by comorbidity with the highest found for ‘diabetes’ (87.5%) and ‘high blood pressure’ (74.3%) and lowest for ‘kidney disease’ (18.8%) and ‘leg pain due to poor circulation’ (26.1%). Sensitivity was increased for comorbidities that were given as specific examples in the questionnaire (e.g. ‘parkinson’s disease’ (65.6%) and ‘multiple sclerosis’ (69.5%), compared to ‘diseases of the nervous system’ (20.9%)).

**Conclusions**

Patient-report appears to provide a reasonable estimate of comorbidity only if comorbid condition categories are clearly defined and reflect a specific clinical manifestation. Such patient questionnaires need to be validated before they are used for research and service evaluation projects.

### O13 Challenges of developing and testing a condition-specific Patient Reported Outcome Measure in a rare condition - Idiopathic Pulmonary Fibrosis (IPF-PRoM Study)

#### Anne Marie Russell^1^, Lesley Anne Saketkoo^2^, Georgina Jones^3^, Melissa Wickremasinghe^4^, Zoe Borril^5^, Sophie Fletcher^6^, Huzaifa Adamali^7^, Toby Maher^8^, Sharon Fleming^8^, Paul Cullinan^1^

##### ^1^National Heart and Lung Institute Imperial College, London, United Kingdom; ^2^Tulane University, New Orleans, LA, USA; ^3^Leeds Beckett University, Leeds, United Kingdom; ^4^Imperial College Healthcare Trust, London, United Kingdom; ^5^Pennine Acute NHS Hospitals Trust, Manchester, United Kingdom; ^6^University Southampton Hospitals Trust, Southampton, United Kingdom; ^7^North Bristol NHS Hospitals Trust, Bristol, United Kingdom; ^8^Royal Brompton Hospital, London, United Kingdom

**Background**

The Idiopathic Pulmonary Fibrosis (IPF) Patient Reported Outcome Measure (PROM) was developed using classical test theory and patient-centred methodology concordant with EMA and FDA criteria [1] and UK NICE guidelines [2]. IPF is a rare restrictive lung condition characterised by significant morbidity and mortality. Generic measures applied in clinical research lack sensitivity. Research activity in IPF is prolific. The absence of a condition-specific measure was perceived to hinder progress.

**Methodology**

Mixed-methods approach. 265 patients contributed to the iterative stages of development supported by a patient, caregiver and professional steering group. 85 patients at 5 UK NHS centres completed the 12-item IPF-PROM to test its reliability. 85 patients continued into the validation study completing the mMRC scale; EQ5-D-5L; IPF-PROM and FVC at three monthly intervals. Twenty patients are completing weekly FVC measurements using hand-held spirometer with telephone support. Nine patients are using an IPF-App.

**Results**

The IPF-PROM has good test-retest reliability (total score t-statistic 0.275; p-value 0.784; ICC 9.24). The mean timeframe TP1–TP2 was 20.69 days. Validation study baseline characteristics: 85 participants; male n= 68 (80%); mean mMRC breathlessness score 1.95 (±1.18). EQ5D domains: mobility 2.43 (±1.21); self-care 1.75 (±1.07); usual activities 1.57 (±1.18); discomfort/pain 2.19 (±1.13); anxiety/discomfort 1.98 (±1.04); VAS score of health today 56.18 (±25.78). FVC 2.58 (±0.65) FVC %predicted 63.38 (±31.77). The mean global health score for the IPF-PROM was 2.84 (±0.81); domain1 7.47 (±2.27); domain2 7.66 (±2.70); domain3 6.49 (± 2.37) domain4 7.42 (±2.30) and total scores 29.05 (±8.61). Total IPF-PROM scores correlated strongly with MMRC (Rˢ0.701 p=0.00001); and EQ-5D Self-care domain (Rˢ0.299 p=0.005).

**Discussion**

The IPF-PROM is a short easy to use questionnaire developed with patient-centred methodology. Further analysis of longitudinal data will explore its psychometric resilience. This work is supported by a NIHR grant.

References

1 www.fda.gov/downloads/Drugs/Guidances/UCM193282.pdf

2 www.nice.org.uk/guidance/cg163

## Theme: Cutting edge methods

### O14 A novel way of anchoring discrete choice experiments valuing EQ-5D

#### Edward Webb^1^, John O'Dwyer^1^, David Meads^1^, Paul Kind^1,2^, Penny Wright^1^

##### ^1^University of Leeds, Leeds, United Kingdom; ^2^University of York, York, United Kingdom

Background

Recently there has been interest in using discrete choice experiments (DCEs) to elicit health state preferences However, results are on a latent scale and require additional information to anchor them to a dead=0, full health=1 scale.

Aim

To investigate a novel anchoring technique requiring only three Visual Analogue Scale (VAS) tasks.

Methods

516 members of the general public completed online DCE questions in which they chose between EQ-5D-3L states, plus one question which offered a dominant option. They rated the states 11111, 33333 and dead using a VAS. Results were analysed using mixed logit models estimated with simulated maximum likelihood (SML) Coefficients were anchored to a 0-1 scale using VAS responses. The effects of removing various participants from the sample were examined. Estimation using SML was compared to estimation using hierarchical Bayes (HB).

Results

After removing respondents who gave illogical VAS responses or “straight lined” answers, N=458 responses were analysed. Coefficients were in the expected direction and statistically significant. Valuations were typically higher than the standard UK EQ-5D-3L tariff. Removing participants who chose a dominated health state resulted in coefficients with a magnitude of 90-115% of the coefficients from the full sample, although differences were not statistically significant. Removing participants who stated they did not understand the VAS task meant coefficients with 80-90% of the magnitude of those from the full sample, though differences were insignificant. Excluding participants rating death above 50 on the VAS gave significantly different coefficients, with magnitudes of 65-70% compared to the full sample, though little additional change occurred from removing participants rating death above 10. Valuations from HB estimation were typically closer to standard tariff values than those from SML.

**Conclusion**

We present an effective way of anchoring DCE results to a QALY scale with little additional respondent burden.

### O15 The “cost” of care for the elderly: differential health status in adult carers and non-carers

#### Francesca Torelli^1^, Paul Kind^1^, David Meads^1^, Penny Wright^2^

##### ^1^Leeds Institute for Health Sciences, University of Leeds, Leeds, United Kingdom; ^2^Leeds Institute for Cancer and Pathology, University of Leeds, Leeds, United Kingdom

**Background**

It is generally accepted that carers of disabled or chronically ill individuals are likely to experience health detriments associated with their role, with that deficit being functionally related to the complexity and demands of the caring task. Little is known about the magnitude of such differential health status and the potential underestimation of outcomes resulting from health and social care interventions.

**Methods**

Health Survey for England (2011 and 2012) include self-reported health as measured using EQ-5D-3L, a generic measure of health status that records problems on 5 health dimensions and a global rating of health on a 0-100 Visual Analogue Scale (VAS). Carer status is also reported in this survey and was used to match caregivers of elderly people with non-carers sharing similar characteristics including age, gender, education and employment status. The impact of socio-demographic variables and burden of care (hours of help/week) was analysed.

**Results**

4,558 individuals were included in the study (mean age=56 years; 60% females). Carers tended to assess their health status more highly (mean VAS = 76.90) than non-carers (mean VAS =75.53). The mean difference in VAS (1.97) was larger in male carers and increased with age (2.89 in the oldest carers). The mean VAS was lower in individuals caring for 20+ hours/week, equal to 72.73.

**Conclusion**

These results indicate that carers appear to assess their health status as marginally better than non-carers, potentially reflecting an intrinsic resilience not hitherto quantified. As the carer task increases in complexity however, self-assessed health decreases. Whilst differences in VAS score were small, the pattern across subgroups showed a clear trend between being a carer of an elderly person and having a higher self-reported health.

### O16 Four short measures for evaluating digital health innovation

#### Tim Benson

##### R-Outcomes Ltd, Thatcham, United Kingdom

Understanding how and why healthcare innovations spread is a key evaluation task.

We could not find short simple survey tools to meet our evaluation needs. As a result, we have developed four related measures, based on practical experience of PROMs and PREMs and understanding of the innovation literature:

- **Innovation Readiness Score** rates how much users are open to new ideas and up-to-date with them, and organisations’ receptiveness and capability to innovate, based on Rogers’ categories of innovator, early adopter, early majority etc. [1].
- **Innovation Adoption Score**, based on May’s Normalisation Process Theory (NPT), to rate the coherence of the process and reflective thought before, during and after implementation [2].
- **Digital Confidence Score**, to rate user’s digital literacy and confidence to use digital products, with dimensions of familiarity, social pressure, support and digital self-efficacy.
- **Application Rating Questionnaire**, to rate each user’s assessment of a specific digital product, as a combination of usefulness, ease of use, support and satisfaction.

These measures share the look and feel of R-Outcomes family of short generic user-reported outcome measures, with a strong family resemblance, being short with a low reading age and generic, applicable to all people and all digital health innovations [3]. Each measure has four questions items and four response options (strongly agree, agree, neutral and disagree). These are labelled, color-coded and use emoji. Mean scores use 0-100 scale for individual items and summary scores.

These measures can be used by patients and staff. One application is the evaluation of the roll out of mobile ECGs to detect atrial fibrillation (AF) to help reduce the incidence of strokes.

References

1. Rogers, Everett. *Diffusion of Innovations* (5^th^ edition). Free Press 2003.

2. May CR, et al. Development of a theory of implementation and integration: Normalization Process Theory. *Implementation Science* 2009; 4(1): 29.

3. http://r-outcomes.com

### O17 Assessing the impact of patient-reported outcome (PRO) data from clinical trials: a systematic review

#### Samantha Cruz^1^, Derek Kyte^1^, Anita Slade^1^, Olalekan Lee Aiyegbusi^1^, Laura Jones^2^, Christell McMullan^2^

##### ^1^Centre for Patient Reported Outcomes Research, Institute of Applied Health Research, University of Birmingham, Birmingham, United Kingdom; ^2^Institute of Applied Health Research, University of Birmingham, Birmingham, United Kingdom

Background

Patient-reported outcomes (PROs) are increasingly collected in clinical trials to provide the patient perspective on the impact of a disease and its treatment. PRO data, if collected, conducted, analysed and reported appropriately, should play a key part in informing patient-centered care and health policy. However, the extent of current PRO impact, barriers and facilitators of impact and metrics to assess PRO specific impact are currently unclear. The aim of this study was 1) to conduct a systematic review to identify: i) common types of research impact associated with PRO trial data, ii) existing metrics used to measure PRO impact and iii) factors that may affect PRO-related impact; 2) to assess REF2014 impact case studies to explore real-world evidence of PRO data impact.

**Methods and findings**

Two independent investigators systematically searched MEDLINE, EMBASE, CINAHL+ and the HMIC databases from inception until January 2018 for publications that discussed the impact of PROs upon clinical decision-making, pharmaceutical labeling claims, clinical guidelines and healthcare policy development. Additionally, the REF2014 database was systematically searched for case studies that included clinical trials collecting PROs. Thirty-eight publications and sixty-nine impact case studies met the inclusion criteria. Nine types of PRO impact were identified through the systematic review. Only 34% of the REF2014 trials collecting PROs led to measurable PRO-related impact. Direct attribution of impact to PRO trial data was possible in twelve trials. In addition, several barriers to maximise PRO trial impact were identified.

**Conclusions**

Measuring PRO impact is an important adjunct to maximising the benefits for patients and society; however, this is a challenging task as PRO data is commonly combined with different clinical outcomes. The implementation of PROs into practice faces a number of barriers, which limit their effective dissemination and impact upon healthcare decisions and patient care. Greater consideration should be given to developing pathways to capture PRO impact by utilising facilitators and removing barriers to implementing PRO impact.

## Theme: Pushing boundaries

### O18 Embedding patient and public involvement within the Centre for Patient Reported Outcomes Research

#### Grace Turner^1^, Olalekan Lee Aiyegbusi^1^, Derek Kyte^1^, Anita Slade^1^, Magdalena Skrybant^1^, Gary Price^2^, Melanie Calvert^1^

##### ^1^University of Birmingham, Birmingham, United Kingdom; ^2^Patient partner, Birmingham, United Kingdom

**Background**

The Centre for Patient Reported Outcomes (PRO) Research (CPROR) at the University of Birmingham aims to optimise use of PROs in clinical trials and routine clinical care, to improve service delivery, enhance patient care and outcomes and ensure the patient perspective is at the heart of health research and decision making. The core ethos of the Centre is “making patient centred care a reality”; therefore, embedding patient and public involvement (PPI) is key to achieve CPROR’s aims.

**PPI within CPROR**

Patient partners were involved in the establishment of the Centre by providing feedback on the CPROR application. At the Centre launch event two patient advocates gave inspiring presentations on the value of PRO research based on their experiences. PPI continues to be embedded in CPROR through representation on the executive committee, which oversees the delivery of the CPROR strategy.

In terms of CPROR research, PPI is incorporated at different stages of the research cycle, including:

1. Research prioritisation;
2. Collaborators on developing research proposals and co-applicants on grant applications;
3. Providing input during the study (such as ethics applications and recruitment strategies);
4. Involvement in dissemination, including being named authors on journal publications.

Specific to PRO research, an example of the value of PPI is during the selection of which PRO measures to include in our research. Patients’ perspectives ensures that the content of PRO measures are appropriate/relevant and the measure is acceptable in terms of questionnaire length, frequency and mode of administration.

**Discussion**

We will present our experiences of embedding PPI within the Centre, aimed at building capacity for PPI in PRO research, both for the research team and patient partners. We will outline the challenges faced and our proposed solutions. We aspire to continue to improve PPI for PRO research and develop best practice.

### O19 An innovative framework for embedding meaningful Patient and Public Involvement in PROMS development

#### Steven Blackburn, Annette Bishop, Krysia Dziedzic

##### Research Institute for Primary Care and Health Sciences, Keele University, Keele, United Kingdom

**Background**

Patient and Public Involvement (PPI) is key to ensuring the relevance, acceptability, and quality of patient-reported outcome measures (PROMs) [1]. Guidance exists on PROMs development, including the use of qualitative research with patients to establish the content and face validity of PROMs [2,3]. However, there is no specific guidance for PPI roles throughout PROMS development. This paper proposes a new framework for embedding meaningful PPI in this process.

Working collaboratively with research team, PPI can have important roles alongside the research activities throughout all stages of PROMs development, as follows:

**Scoping**

*Research:* i) Literature review of existing PROMs and relevant outcomes, ii) Expert opinion

*PPI:* i) Review quality and acceptability of existing PROMs; ii) Identify the need for a new PROM; iii) Advise on research plan (e.g. recruitment, interview topic guide)

**Conceptual Framework & draft PROM**

*Research:* Qualitative interviews with patients to identify important outcomes

*PPI:* i) Conduct interviews; ii) Analyse and interpret findings; iii) Develop conceptual framework; iv) Draft PROM content

**Iterative development**

*Research:* Cognitive interviews to verify the PROM's face and content validity

*PPI:* i) Analyse and interpret findings; ii) Finalise PROM wording and format; iii) Support translation and cultural adaptation for use in other countries

**Assessment of psychometric properties**

*Research:* Observational or experimental study

*PPI:* Interpreting the psychometric properties from patient/public perspective (e.g. missing data, minimal clinical important difference)

**Dissemination and implementation**

*Research:* Publish

*PPI*: i) Support dissemination to the public; ii) Encourage uptake of the PROM in clinical practice

**Conclusion**

A framework for embedding meaningful PPI throughout the PROMS development process is proposed. Previous studies have implemented individual framework elements. Future work will test all elements together to assess added value and impact.

**Acknowledgements**

KD is part-funded by a NIHR Knowledge Mobilisation Research Fellowship (KMRF201403002) and NIHR CLAHRC West Midlands

References

1. Staniszewska, S. Patient.2012;5(2):79-87.

2. www.fda.gov/downloads/Drugs/.../Guidances/UCM193282.pdf

3. https://www.ema.europa.eu/en/appendix-2-guideline-evaluation-anticancer-medicinal-products-man-use-patient-reported-outcome-pro

### O20 Co-constructing a new Axial Spondyloarthritis (axSpA) specific measure of energy and fatigue: a researcher’s reflection on patient involvement

#### Nathan Pearson^1^, Jane Martindale^2,3^, George Strickland^3^, Jean Thompson^3^, Elizabeth Tutton^1,4^, Jonathan Packham^5,6^, Helen Parsons^7^, Kirstie Haywood^1^

##### ^1^Warwick Research in Nursing, Warwick Medical School, University of Warwick, Coventry, United Kingdom; ^2^Faculty of Health and Medicine, Lancaster University, Lancashire, United Kingdom; ^3^Wrightington, Wigan and Leigh NHS Foundation Trust, Wigan, United Kingdom; ^4^Trauma Research, Oxford University Hospitals NHS Foundation Trust, Kadoorie Centre, John Radcliffe Hospital, Oxford, United Kingdom; ^5^Institute of Applied Clinical Science, Keele University, Staffordshire, United Kingdom; ^6^Haywood Academic Rheumatology Centre, Staffordshire, United Kingdom; ^7^Clinical Trials Unit, Warwick Medical School, University of Warwick, Coventry, United Kingdom

Aim

A reflection on the active collaboration with patient research partners (PRP) in the co-construction of a new measure.

Methods

The Guidance for Reporting Involvement of Patients and the Public – version 2 short-form (GRIPP2-SF) was applied retrospectively to assist with the reflection. Initial measurement development involved a range of qualitative approaches, generating a long-form questionnaire ready for psychometric testing. Five members of an established PRP group were involved throughout the project, including: the co-construction and piloting of study materials, data interpretation, discussions leading to the development of an axSpA-specific conceptual framework of energy and fatigue, and reviewing potential items. Collaborative activities included: 1) Semi-structured group meetings at key stages, co-facilitated by two non-patient researchers (NP, JM). Physical documents were mailed to participants; 2) E-mail dissemination of meeting summaries and follow-up on issues raised.

**Results**

Four group meetings were held– each with between three and five PRP attendees. No PRP attended all meetings. The PRPs contributed in several ways – for example: co-constructing interview topic guides to reflect important facets of fatigue, supporting data interpretation, peer-reviewing the developing conceptual model and item generation, and supporting ethics applications.

**Discussion**

Two PRPs (GS, JM) were involved from study conception, which in collaboration with the research team facilitated the contribution of PRPs throughout the study. PRP funding was factored into the grant application. All PRPs influenced important aspects of the study, which may have been supported by receiving formal training sessions in advance of the study, supplemented by access to a webpage and video link. Additionally, all members had a pre-existing relationship with the group facilitator (JM) which, coupled with a welcoming and supportive environment enabled the active embedding of PRPs throughout the study. However, challenges included a changing PRP membership and inconsistent attendance at group meetings, resulting in a diminished group collective knowledge.

## Theme: Economic evaluation

### O21 Time Trade-Off (TTO) – does there need to be clarity on how health state descriptions are developed and the task administered?

#### Charlotte E Kosmas, Samuel Llewellyn, Helen Doll

##### ICON plc, Abingdon, United Kingdom

Time Trade-Off (TTO) methodology is a standard approach that is used to assess quality of life (QoL) by asking individuals in a valuation exercise to what extent they would trade (lose) years of life in exchange for improved QoL. The different approaches to TTO methodology and its limitations have been well documented, all of which have important implications for the utility values generated for economic evaluation. Despite this, the TTO approach remains widely accepted and is the preferred technique cited in the NICE Methods Guidance as an alternative to collecting EQ-5D data when such data are not available or appropriate. Little attention has focused on methods of developing the health state descriptions for TTO studies and on different procedural considerations for administering the task. As the health state descriptions (vignettes) are a key component of the TTO exercise, following a comprehensive and rigorous approach to developing the vignettes is central to the valuation exercise and thus the utility values derived from them. In this work the authors describe an approach to developing vignettes for use in the general public. The importance of following key steps such as conducting a targeted literature review to understand the nature of the condition of interest, involving patients and clinical experts in vignette development, as well as the need to pilot the vignettes in a cognitive interview are discussed. The authors also highlight different procedural considerations for conducting a TTO study such as participant recruitment and outline potential issues that can arise during the interview process itself. These considerations will allow future researchers to reflect on the most appropriate approach to achieve optimal study design.

### O22 Valuing the EQ-5D-Y using a discrete choice experiment: do adult and adolescent preferences differ?

#### David Mott^1^, Koonal Shah^1^, Oliver Rivero-Arias^2^, Juan Manuel Ramos-Goñi^3^, Nancy Devlin^1^

##### ^1^Office of Health Economics, London, United Kingdom; ^2^University of Oxford, Oxford, United Kingdom; ^3^EuroQol Research Foundation, Rotterdam, Netherlands

The EQ-5D-Y is a patient reported outcome measure that is used to capture health states experienced by children and adolescents. Currently, no tariffs exist to assign utilities to EQ-5D-Y health states for economic evaluations. In their absence, tariffs for a different instrument (i.e. EQ-5D-3L) have been applied to generate utilities.

One of the challenges associated with valuing the EQ-5D-Y is that standard valuation methods can be cognitively demanding for adults and may therefore be even more challenging for younger individuals. However, adults may find it difficult to complete a valuation task from the perspective of a child or an adolescent. Therefore it may be desirable to seek the views of adolescents directly, using a methodology that is easier for young people to understand compared with traditional methods.

This paper has two aims. The first is to elicit and analyse latent scale discrete choice experiment (DCE) valuation data that could be used to generate an EQ-5D-Y value set for the UK. The second is to evaluate whether there are systematic differences in the preferences obtained from adolescents and those obtained from adults considering the health of a child.

An online survey was designed containing a DCE as well as additional background and feedback questions. The DCE comprised 15 pairwise choices, where the alternatives were different EQ-5D-Y health states. A representative sample of 1,000 UK adults received a DCE framed such that the respondents should consider the health of a 10-year-old child when completing the tasks. In contrast, a sample of 1,000 UK adolescents (11-to-17 years) received the same survey but was asked to consider their own health.

Data collection has recently been completed and analysis is on-going, utilising a range of flexible choice models. The paper will present the results and consider the implications of the findings for research and policy.

## Theme: Digital Capture of PROs

### O23 eRAPID: electronic patient self-Reporting of Adverse-events: Patient Information and aDvice - patient recruitment and retention in pilot and randomised studies in oncology

#### Galina Velikova^1^, Kate Absolom^1^, Simon Pini^1^, Trish Holch^2^, Lorraine Warrington^1^, Marie Holmes^1^, Andrea Gibson^1^, Zoe Rogers^1^, Sarah Dikinson^1^, Robert Carter^1^, Beverley Clayton^1^, Susan Davidson^3^, Jacqueline Routledge^3^, Kevin Franks^1^, Ann Henry^1^

##### ^1^University of Leeds, Leeds, United Kingdom; ^2^Leeds Beckett University, Leeds, United Kingdom; ^3^The Christie Hospital, Manchester, United Kingdom

**Background**

eRAPID is an online system for patients to self-report symptoms and adverse events (AE) during and after cancer treatments. Patient reports are integrated into Electronic-Patient-Records to be used in patient care. The system provides patient advice for mild AE and notifications to clinicians for severe AE. The goal of eRAPID is to improve the safety of cancer treatments and enhance patient care.

**Methods**

eRAPID is evaluated in pilot and randomized studies:

A randomized-controlled-trial (RCT) evaluates patient benefits and cost-effectiveness of eRAPID during chemotherapy for breast, ovarian and colorectal cancers. Randomisation is to eRAPID intervention (weekly online AE reporting with feedback for 18 weeks) or usual care. Outcome measures include patient quality-of-life, self-efficacy, impact on process of care (acute admissions, telephone calls, unplanned appointments).

A two-centre pilot study in pelvic radiotherapy for prostate, gynaecological and lower gastro-intestinal cancers uses eRAPID to monitor AE during and up-to 6 months after treatment.

Here, we report patient recruitment and retention into the above studies.

**Results**

Since May-2016 the RCT recruited 369 patients (target sample n=419), 149 patients declined (consent rate 71%). Further 57 patients were identified but on the 2^nd^ screening were ineligible due to not using Internet or not having chemotherapy; 122 patients were not eligible-lack of Internet access. Retention rates are good, only 10% left the trial: 23 became too ill/disease progression and 15 patients actively withdrew. Reason for withdrawal: Not enough time n=5; feeling ill n=3, too stressful n=5.

Since December 2016 the pilot radiotherapy study recruited 157 (target n=168 patients), 47 patient declined (consent rate 78%), 14 patients withdrew (9%).

**Conclusions**

Recruitment figures suggest this approach is feasible and acceptable to the majority of cancer patients. A sizable patient minority cannot use Internet for home-reporting. Recruitment finishes May-2018 with analysis November-2018.

*This is independent research funded by National-Institute-for-Health-Research (RP-PG-0611-20008). The views are those of the author(s) and not necessarily of NHS, NIHR or Department-of-Health.*

### O24 Feasibility of digital self-report PRO data for monitoring adverse events after discharge following major abdominal cancer surgery: the eRAPID study

#### Kerry Avery^1^, Hollie Richards^1^, Amanda Portal^1^, Trudy Reed^2^, Ruth Harding^2^, Robert Carter^3^, Kate Absolom^3^, Galina Velikova^3^, Jane Blazeby^1^

##### ^1^University of Bristol, Bristol, United Kingdom; ^2^University Hospitals Bristol NHS Foundation Trust, Bristol, United Kingdom; ^3^University of Leeds, Leeds, United Kingdom

**Aims**

Major abdominal cancer surgery can result in adverse effects (AEs). If complications occur at home, late detection and treatment may lead to increased emergency admissions. Prompt identification of AEs is important to improve patient safety and outcomes. eRAPID is an online PRO platform, integrated within hospital electronic patient records (EPR), for patients to report PROs during recovery, in which tailored self-management feedback or advice to contact a clinician is generated. Clinicians are alerted to symptoms indicative of AEs by email. This prospective feasibility study examines the functionality and feasibility of eRAPID to support patient PRO self-tracking during recovery and inform clinical decision-making.

**Methods**

47 patients (30 men, mean age 62.2) undergoing major abdominal cancer surgery (e.g. gastrectomy, hepatectomy) were invited to complete the online eRAPID symptom questionnaire pre-discharge and a minimum of 9 times up to 8-weeks post-discharge. Patients complete 37 questionnaire items regarding 24 symptoms. Initial analyses explored the system’s functionality, data completeness and stratification of potential AEs.

**Results**

23 patients (49%, 16 men, mean age 64.7) consented to participate and completed the questionnaire a total of 163 times. Of these, 21 (91%) completed the questionnaire pre-discharge and 16 (70%) ≥7 times post-discharge. Response rates were lowest (range 48-52%) from week 7 post-discharge. Of 163 completions, 13 (8%) triggered AE alerts to clinicians, 69 (42%) triggered advice to contact clinicians regarding symptoms and 63 (39%) triggered patient self-management advice for expected symptoms.

**Conclusion**

A system for digital PRO data collection post-discharge following surgery has been developed and integrated into EPR. Preliminary findings indicate the eRAPID system is acceptable for patients to complete and can tailor clinician alerts and patient self-management advice dependent on the severity of symptoms reported. Further analyses will establish actions/outcomes resulting from AE alerts to clinicians and advice for patients to contact clinicians.

### O25 Assessment of measurement equivalence of the paper-based and electronic version of a newly developed haematology specific patient-reported outcome measure, HM-PRO

#### Pushpendra Goswami^1^, Tatiana Ionova^2^, Esther Oliva^3^, MS Salek^1^

##### ^1^University of Hertfordshire, Hatfield, United Kingdom; ^2^St. Petersburg State University and Multinational Centre for Quality of Life Research, St. Petersburg, Russian Federation; ^3^Haematology Unit, Grande Ospedale Metropolitano, Reggio Calabria, Italy

**Aims**

To assess equivalence of the paper-based and the electronic application of the newly developed HM-PRO.

**Methods**

Following International Society for Pharmacoeconomics and Outcomes Research (ISPOR) ePRO Guidelines *on evidence needed to support measurement equivalence between electronic and paper-based PRO*, 193 adult patients (62% male, median age 66.5 year, IQR 17.9 – 89.1) with different Haematological malignancy (HM) (ALL-12; AML-28; CLL-17; CML-13; MM-33; INHL-16; ANHL-22; HL-14; MDS-15; and MPN- 23) were recruited into a UK multi-centre (i.e. 7 secondary care hospitals) prospective study. The median time since diagnosis was 1.7 years (IQR 0.002-25.8). Both Paper-based and electronic version of the HM-PRO were completed by patients in a randomized crossover design. The effects of instrument version and order was assessed using a 2-way ANOVA test. Intra-class correlation and Spearman’s rank correlation coefficients were used to evaluate reproducibility/test-retest reliability. In addition, 10 patients were randomly selected for cognitive interviews.

**Results**

The questionnaire version and administration order effects were not significant at the 5% level. Further, no interaction was found between these two factors for both parts of HM-PRO (PART A (QoL), P=0.95); and Part B (signs & symptoms), P=0.72). Spearman’s rank correlation coefficients were greater than 0.9, and ICC ranged from 0.94 – 0.98; and score were not statistically different between the two versions, showing acceptable reliability indexes. Moreover, the difference between the completion time for both paper (mean 6:38 min) and electronic version (mean 7:29 min) was not statistically significant (n=100, P=0.11). Patients did not report any difficulty in completing the electronic version during cognitive interviews, and were able to understand and respond spontaneously.

**Conclusion**

Scores for electronic and paper-based versions for both parts of HM-PRO were comparable and difference was not statistically significant. The electronic version shows good reliability and face validity which required minor modification to the original paper version fulfilling ISPOR ePRO Task Force Guidelines.

### O26 Thrive - online development of a positive and patient-centric PROM to support self-management and biomedical discovery on PatientsLikeMe

#### Paul Wicks, Stacey McCaffrey, Kim Goodwin, Ryan Black, Jamie Harisiades, Michael Hoole, James Heywood

##### PatientsLikeMe, Cambridge, MA, USA

**Background**

Since 2006, >625,000 members of PatientsLikeMe (PLM) have completed PROMs in conditions such as ALS/MND, Parkinson’s, and mood disorders. Reflecting back PROM scores with context helps members find “patients like them” and improve self-management. However, traditional PROMs have a number of limitations; most were not designed to be reflected to patients, burden increases dramatically with comorbidity, item phrasing is generally negative, and there is variation in psychometric quality.

**Objective**

We sought to develop a new universal, positively framed, and modular measurement system, named “Thrive” to leverage our members’ increasing use of mobile and machine learning for multi-omic discovery research.

**Methods**

A conceptual framework was combined with data from ~20 existing PROMs on PLM to generate initial items, which underwent several rounds of cognitive debriefing. An online survey tool fielded a revised set of items alongside the SF-20 and PHQ-9 to nearly 2,000 PLM members with a range of chronic health conditions. Following psychometric evaluation, items were revised and fielded to another 700 PLM members.

**Results**

The finalized instrument consists of a 21-item core including three multi-item subscales; “Core symptoms”, “Abilities”, and “Thriving.” Results provide evidence of content and construct (convergent, discriminant) validity, high levels of test-retest and internal consistency reliability, and ability to detect change over time. The items did not exhibit substantial bias based on gender, race, or condition. These results support the use of Thrive across diverse patient populations.

**Discussion**

Thrive appears a useful way of consolidating important domains for patients with chronic conditions. This “core” set serves as a foundation to begin developing modular condition-specific versions in the future. Cross-walking against traditional PROMs from the PLM platform is underway, in addition to clinical validation. Thrive is licensed under Creative Commons Sharealike.

### O27 Do patients engage with an online patient reported outcome measure (PROM), post-shoulder surgery, and what factors influence their engagement?

#### Alice Bellchambers^1^, Cameron Hatrick^2^

##### ^1^Brighton and Sussex Medical School, Brighton, United Kingdom; ^2^Brighton and Sussex University Hospitals NHS Trust, Brighton, United Kingdom

**Aim**

To evaluate engagement with an online Patient Reported Outcome Measure (PROM) post-shoulder surgery, in order to ensure that PROM collection, particularly the use of online PROMs, is optimised within the clinical context of shoulder surgery.

**Methods**

486 patients who had undergone shoulder surgery by the same surgeon between 2013 and 2016, and who had completed a paper-copy Oxford Shoulder Score (OSS) pre-operatively, were followed up by telephone interview. Statistical analyses were performed to compare those who had tried to use, and those who had successfully used the online PROM (MyClinicalOutcomes), with patients who did not engage with the website, in order to identify variables associated with engagement. Reasons for disengagement were analysed, as were patients’ opinions of the website.

**Results**

Post-operative OSSs were collected for 72.2% of operations; 26% of these were completed online and the remaining 74% were collected via telephone. 37% of patients who were successfully followed up via telephone reported that they had tried to use MyClinicalOutcomes, with 77% managing to successfully complete a post-operative OSS on the website. The main reason given for not trying to use MyClinicalOutcomes was that patients were unaware of it. Those under the age of 40 were significantly less likely to try to use, or to successfully use, MyClinicalOutcomes, than those over 40.

**Conclusion**

Patient engagement with MyClinicalOutcomes was poor, adding further evidence to the current literature, which suggests online methods of PROM collection may not currently yield sufficient response rates for their widespread implementation, and that mixed methods should be used in the meantime. The main reason for disengagement was that patients were unaware of MyClinicalOutcomes, suggesting better provision of information about the tool may be necessary in the future. A relatively weak association was found between younger patients and disengagement with MyClinicalOutcomes, which may warrant further investigation.

### O28 The use of an electronic Patient-Reported Outcome Measure in the management of patients with Advanced Chronic Kidney Disease: the RePROM Pilot Trial

#### Derek Kyte^1^, Jon Bishop^2^, Elizabeth Brettell^2^, Melanie Calvert^1^, Paul Cockwell^3^, Mary Dutton^3^, Helen Eddington^3^, Gabby Hadley^3^, Natalie Ives^2^, Louise Jackson^4^, Stephanie Stringer^3^, Marie Valente^2^

##### ^1^Centre for Patient-Reported Outcomes Research, University of Birmingham, Birmingham, United Kingdom; ^2^Birmingham Clinical Trials Unit, University of Birmingham, Birmingham, United Kingdom; ^3^University Hospitals NHS Foundation Trust, Birmingham, United Kingdom; ^4^University of Birmingham, Birmingham, United Kingdom

**Background**

Chronic Kidney Disease (CKD) affects 1 in 7 people in the UK. Those progressing to end stage renal disease (ESRD) experience significant symptom burden, reduced quality of life and increased hospitalisation and mortality. Accurate and responsive healthcare for patients moving from advanced CKD to ESRD is therefore a key healthcare priority.

A recent US oncology RCT demonstrated that routine patient use of an electronic Patient-Reported Outcome Measure (ePROM), which provided real-time data to clinicians, was associated with: improved quality of life; reduced A&E visits and hospitalisations; and superior survival. The value of ePROMs has not been tested in CKD. Therefore, a RCT is needed to assess whether ePROM use can improve patient outcomes compared to standard treatment alone. Before this can happen, a pilot trial is needed to determine if a RCT can be conducted.

**Methods**

Stage 1 will involve the development of a new kidney ePROM system, which will allow: (i) patients to input their data in multiple ways (e.g. via computer or smartphone or tablet) and (ii) clinicians to view the patient’s data in real-time.

In stage 2, a pilot study will be conducted in which 66 consenting patients with advanced CKD will be randomly allocated to receive either standard treatment alone or standard treatment plus the ePROM. The study will assess: (a) the willingness of clinicians/patients to be involved in the study; (b) acceptability of the ePROMs; and (c) which is the most appropriate primary outcome to measure the efficacy of the ePROM intervention in the full-scale RCT.

**Discussion**

Routine clinical use of ePROMs may aid patient self-management, improve the flow of information between patients and their clinicians and optimise patient safety and outcomes. We will present an update on initial findings of the RePROM pilot trial, due to start recruitment in April 2018.

### O29 Increasing capture of patient-reported outcomes in trauma research

#### Grace Turner^1^, Ameeta Retzer^1^, Anita Slade^1^, Derek Kyte^1^, Karen Piper^2^, Tony Belli^1,2^, Melanie Calvert^1^

##### ^1^University of Birmingham, Birmingham, United Kingdom; ^2^Queen Elizabeth Hospital Birmingham, Birmingham, United Kingdom

**Background**

In order to understand how peoples’ quality of life is affected following major trauma and the effects of that injury on their health and wellbeing, it is important to capture patients’ perspectives of their own health. Patient Reported Outcome Measures (PROMs) can capture patients’ own experience of their health such as symptoms, mobility, mental health and social function.

The aim of this research is to establish the impact of trauma on quality of life/ symptoms and to explore views on using PROMs to support clinical care and research.

**Methods**

One-to-one, semi-structured interviews will be conducted with: (i) people who have experienced a major trauma, (ii) their family members/ carers; (iii) healthcare professionals working in trauma related clinical areas; (iv) trauma researchers; and (v) staff members/ volunteers from third sector organisations who support trauma patients and their families/carers.

**Results**

This is an ongoing study based at the Centre for Patient Reported Outcomes Research (CPROR) funded by the National Institute for Health Research Surgical Reconstruction and Microbiology Research Centre (NIHR SRMRC).

Findings will be used to inform the development of a pathway for the electronic capture of PROMs for inclusion within routine clinical care of trauma patients and trauma research. Future research will test the feasibility and acceptability of this ePROM system.

The research programme is being delivered in close collaboration with key stakeholders, including patient partners, trauma clinicians, trauma researchers and the ministry of defence.

**Conclusion**

The rising number of major trauma survivors has driven the need for improvements in rehabilitation to enable patients to return to functional activities, work and education after complex re-enablement and reconstructive surgery. PROMs are essential to deliver patient-centred healthcare and research which is informed by patient-focused priorities/outcomes. The programme will also increase capacity for trauma-specific knowledge and expertise in relation to PROMs.

### O30 A qualitative study of patients’ and clinicians’ perspectives on the use of electronic Patient-Reported Outcome measures (ePROMs) in the management of patients with Advanced Chronic Kidney Disease (PRO-trACK Project)

#### Olalekan Lee Aiyegbusi^1,2^, Derek Kyte^1,2^, Paul Cockwell^1,3^, Tom Marshall^1,2^, Mary Dutton^3^, Natalie Walmsley-Allen^3^, Ram Auti^4^, Melanie Calvert^1,2^

##### ^1^Centre for Patient-Reported Outcomes Research, University of Birmingham, Birmingham, United Kingdom; ^2^Institute of Applied Health Research, University of Birmingham, Birmingham, United Kingdom; ^3^Department of Renal Medicine, University Hospitals Birmingham NHS Foundation Trust, Queen Elizabeth Hospital Birmingham, Birmingham, United Kingdom; ^4^Information Technology Services, University Hospitals Birmingham NHS Foundation Trust, Queen Elizabeth Hospital Birmingham, Birmingham, United Kingdom

**Background**

Chronic kidney disease (CKD) can significantly affect patients’ health-related quality of life (HRQOL). Electronic patient-reported outcome measures (ePROMs) could be used to capture patients’ symptoms/HRQOL and assist clinicians with the management of patients with advanced CKD.

**Aim**

To explore patient and clinician views on the use of a renal ePROM system incorporating electronic versions of the Kidney Disease Quality of Life-36 (KDQOL-36) or the Integrated Patient Outcome Scale-Renal (IPOS-Renal).

Methods and analysis: 12 semi-structured face-to-face/telephone interviews were conducted with CKD patients; a focus group (eight participants) and 12 face-to-face semi-structured interviews were conducted with renal staff (doctors, nurses and other allied health professionals) from University Hospitals Birmingham NHS Foundation Trust. The discussions were audio recorded and transcribed verbatim. Transcripts were coded using the Nvivo 11 Plus software. Thematic analysis was conducted to identify the main themes.

**Results**

Patients reported: (1) a favourable assessment of the content validity of the two questionnaires; (2) that the implementation of a renal ePROM system would be acceptable and potentially beneficial in their care; (3) a willingness to complete ePROMs on a regular basis despite clinician concerns about patient burden; and (4) a desire for feedback from their clinicians. Clinicians expressed an interest in the routine use of a renal ePROM system but raised concerns about its potential impact on current clinical practice such as: (1) increasing their workload; (2) unrealistically raising patient expectations; and (3) medico-legal issues. Patients and clinicians identified potential benefits, barriers and facilitators for the use of a renal ePROM system in routine care.

**Conclusion**

Patients generally welcomed the idea of using a renal ePROM system as part of their care. Clinicians acknowledged that a renal ePROM system could potentially have an important supportive role in the management of patients with CKD in routine clinical care.

## Theme: Cutting edge methods

### O31 What outcome measures should we use with informal carers? An analysis of the validity of 5 different measures across 4 conditions

#### Carol McLoughlin, Ilias Goranitis, Hareth Al-Janabi

##### University of Birmingham, Birmingham, United Kingdom

**Background**

Carer quality of life (QoL) effects are recommended for inclusion in economic evaluations and are often used in evaluative research. However, no head-to-head comparison has been done of the relative performance of different types of QoL outcome measures for informal carers.

**Aims**

This study compared the construct validity of three ‘care-related’ QoL measures (the Carer Experience Scale (CES), CarerQoL, and ASCOT-Carer), and two generic QoL measures (the EQ-5D-5L and ICECAP-A) in a UK sample of informal carers of adults suffering from dementia, stroke, mental illness or rheumatoid arthritis.

**Methods**

A questionnaire containing the five QoL measures and additional questions related to the carer, care recipient and the caring situation was posted to eligible carers identified through the Family Resources Survey (n=1,004). Hypotheses regarding the anticipated associations between constructs related to the QoL of carers were developed. Construct validity was assessed by testing associations between measure scores and contextual constructs for each condition, and condition-specific health difficulties.

**Results**

Each measure exhibited some level of construct validity with 14 of the 16 associations that were hypothesised being statistically significant across all measures in the pooled sample. In the condition specific analyses the ASCOT-Carer had more statistically significant associations than the CES or the CarerQoL. Of the generic measures the ICECAP-A was strongly correlated with the care-related QoL measures. The ASCOT-Carer and ICECAP-A were comparable in detecting larger effect sizes and stronger associations, relative to the other measures, across all conditions.

**Conclusion**

The results of this study provide encouraging initial evidence of the validity of care-related and generic QoL measures in informal carers of adults. Care-related measures were not always more sensitive to constructs associated with QoL of carers compared to generic measures. The performance of the ICECAP-A was comparable to that of the best performing care-related measure, the ASCOT-Carer.

### O32 Development of a quality of life instrument for dementia carers

#### Mike Horton^1^, Molly Megson^1^, Paul Kind^1^, Jan Oyebode^2^, Linda Claire^3^, Hareth Al-Janabi^4^, Carol Brayne^5^, Alan Tennant^6^, Zoe Hoare^7^, Penny Wright^1^

##### ^1^University of Leeds, Leeds, United Kingdom; ^2^University of Bradford, Bradford, United Kingdom; ^3^University of Exeter, Exeter, United Kingdom; ^4^University of Birmingham, Birmingham, United Kingdom; ^5^University of Cambridge, Cambridge, United Kingdom; ^6^Swiss Paraplegic Research, Nottwil, Switzerland; ^7^Bangor University, Bangor, United Kingdom

**Background**

Having a caring role can have a huge impact on Quality of Life (QoL). It is therefore important that QoL is accurately measured so that support can be targeted appropriately. The aim of the DECIDE (Dementia Carers Instrument Development) project is to follow an inductive needs-led approach to QoL, in order to develop a new psychometrically sound, brief instrument to measure the impact of caring for someone living with dementia.

**Methods**

Interviews with 42 carers generated an initial item set of 70 dichotomous (agree/disagree) items relating to their QoL. Psychometric evaluation data were collected in 22 locations across England and Wales, from a range of services and settings, resulting in a useable sample of N=566.

A needs-led approach lends itself to a unidimensional framework, therefore a Rasch analysis was carried out on the entire item set. The intention was to remove individual item-anomalies in order to derive a unidimensional item bank alongside a representative, brief short-form instrument.

**Results**

An initial Rasch analysis of the complete 70-item set revealed extensive misfit and a severe breach of the unidimensionality assumption. An exploratory factor analysis was therefore carried out, which identified four potential factors. All items loading onto the first factor (n=36) were considered as candidates for the primary scale of interest, and these were taken forward into a secondary Rasch analysis. This indicated a number of individual misfit anomalies, which were subsequently removed on an iterative basis, in order of misfit magnitude.

**Conclusion**

A patient-centred approach was used to create a robust 18-item instrument to measure the impact of caring for someone with dementia. This scale is shown to be valid, reliable, unidimensional, free from differential item functioning (item bias) for age, gender and carer-relationship, free from local dependency, and well-targeted to the carer population.

### O33 Feasibility of collecting and assessing patient-reported outcomes for emergency admissions: laparotomy for gastrointestinal conditions

#### Esther Kwong, Jenny Neubuger, Nick Black

##### LSHTM, London, United Kingdom

**Aim**

To assess the feasibility of obtaining emergency surgical patients’ prior health status by recall and their outcome by mailed questionnaire three months after.

**Methods**

Patients undergoing emergency laparotomy for gastrointestinal conditions were recruited in 11 hospitals. Recruitment was assessed by: the proportion of emergency admissions eligible for inclusion; the proportion of patients who were invited to participate; and the proportion who completed a PROM (EQ-5D and Gastrointestinal Quality of Life Index - GIQLI). Response rate at follow-up was measured and response bias determined; outcome was compared with retrospective baseline PROMs using χ²and paired t-test for differences.

**Results**

Most patients 466 (85%) met the eligibility criteria, 395 (84%) were invited to participate of whom 268 (68%) completed a retrospective PROM.

Recruitment metrics varied between hospitals: eligibility 72-97%; invited to participate 60-93%; and agreement to participate 55-92%. While case-mix differences might account for some variation, these findings suggest less well performers could improve their recruitment processes.

Among 255 survivors at three months, 190 patients (74.1%) responded to the follow-up PROM (145 to the first request and 44 after one reminder). Responders were similar to non-responders as regards living arrangements, comorbidities, and generic and disease-specific PROMs. They were, however, more likely to be older, female, and less deprived. Patients’ health had been restored to pre-event levels as regards GIQLI Index (and Emotion and Physical subscales), improved as regards the Symptom sub-scale but deteriorated as regards the Social sub-scale. The EQ-5D-3L had improved (0.58-0.64; p=0.06)

**Conclusion**

It is feasible to collect retrospective PROMs from patients admitted unexpectedly for emergency laparotomy. The generalisability of these findings to other causes of emergency admissions needs to be established. This approach offers the opportunity for assessing, from the patient's perspective, the impact of treatment for the 40% of hospital admissions that are emergencies.

### O34 Digital PRO data collection is a beneficial adjunct to patient self-management during recovery following major abdominal cancer surgery: the eRAPID study

#### Hollie Richards^1^, Kerry Avery^1^, Amanda Portal^1^, Trudy Reed^2^, Ruth Harding^2^, Robert Carter^3^, Kate Absolom^3^, Galina Velikova^3^, Jane Blazeby^1^

##### ^1^University of Bristol, Bristol, United Kingdom; ^2^University Hospitals Bristol NHS Foundation Trust, Bristol, United Kingdom; ^3^University of Leeds, Leeds, United Kingdom

**Background**

Complications after discharge from hospital following surgery are common. Monitoring of adverse events (AEs) during recovery is not standardised. A digital system for patients to self-report symptoms could support patient self-management and inform clinical decision-making. eRAPID is an online PRO platform, integrated within hospital electronic patient records (EPR), for major abdominal surgery patients. Tailored self-management feedback or advice to contact a clinician is generated. Clinical Nurse Specialists (CNS) are also alerted to symptoms indicative of AEs by email. This prospective qualitative study explored patients’ and clinicians’ perspectives of the feasibility of eRAPID to support patient self-management and enhance clinical decision-making.

**Methods**

Major abdominal cancer surgery (e.g. gastrectomy, hepatectomy) patients were recruited at hospital discharge and invited to complete the eRAPID questionnaire weekly for 8 weeks. Telephone interviews were conducted weekly with patients to explore patients’ opinions of the system, the acceptability of and adherence to the advice. CNS were interviewed about their use of the PRO data, the applicability of alerts triggered and how these impacted on clinical decision-making. In this first phase of analyses, targeted transcriptions of audio-recorded interviews were coded using methods of constant comparison.

**Results**

In total, 25 interviews with 12 patients (11 men, mean age 66) and 2 CNS have been analysed to date. Preliminary findings indicate the eRAPID system provides a definitive source of self-management advice. Routine self-reporting of PROs was considered a positive adjunct to patients’ post-operative care, enabling patients to track their progress and providing reassurance. Many of the alerts were considered valuable by clinicians for triaging patients without increasing workload.

**Conclusion**

Digital PRO data collection is a beneficial adjunct to patient self-management during recovery. A system that alerts clinical staff to AEs can enhance patient management and care. The eRAPID system has the potential for widespread application to other disease areas.

